# Adolescent Depression and Cognition Risk for Suicide: An Investigation of Risk Factors and Gene Environment Interactions

**DOI:** 10.1002/brb3.70247

**Published:** 2025-02-05

**Authors:** Stefanie Pilkay, Devorah Halevy, Danielle Femia, Sage Malina Holland, Stephanie Heiland, Andrew M Carroll, Sarah Nunes, Tara Veerman, Leah Dudak

**Affiliations:** ^1^ School of Social Work Syracuse University Syracuse New York USA; ^2^ Wurzweiler School of Social Work Yeshiva University New York New York USA; ^3^ School of Social Work Centenary University Hackettstown New Jersey USA; ^4^ School of Information Studies Syraucse University Syracuse New York USA

## Abstract

**Background:**

Prior research has been conducted to understand the connection between maternal physical health and its effects on children and adolescents. Much less research has focused on maternal mental health and gene‐environmental factors that influence children's and adolescent's development. This study investigated the risk factors and gene‐environment interactions in relation to maternal mental health and adolescent depression and cognition risk for suicide.

**Methods:**

The data and sample utilized in this research were obtained from The Future of Families and Child Wellbeing Study. The cohort comprises a total of 4898 children and their corresponding caregivers, of which 1829 had complete data and were used in this study. To account for missing data, the list wise deletion approach was employed for all analyses. The variables were tested using Likert scales and/or other similar scales designed to specifically measure the variable. Hypotheses were tested with multiple regressions and bootstrapping. Multiple comparisons were controlled with the Benjamini Hochberg false discovery approach, and BH adjusted *p*‐values are reported with each statistically significant result.

**Results:**

The analyses revealed four significant relationships: (1) poverty status is a strong predictor of adolescent mental health, (2) toddler attachment is affected by maternal mental health but does not interact with maternal mental health to predict adolescent mental health, (3) maternal mental health affects suspected childhood sexual abuse associations with adolescent mental health, and (4) serotonin transporter alleles (SLC6A4) exert specific effects on adolescent mental health cognitions previously linked to suicide risk.

**Conclusion:**

In summary, this study concludes that the experiential factors and genetic variation have interconnected influences on the mental well‐being of children and adolescents. Therefore, interventions aimed at addressing child trauma would be enhanced by integrating a component that specifically addresses the mental well‐being of mothers.

## Graphical abstract

Maternal mental health has direct effects on toddler attachment and adolescent mental health and moderates the effects of suspected sexual abuse in childhood on adolescent mental health. The SLC6A4 LL gene variant moderates the effects of maternal mental health on adolescent mental health. Moderating variables are highlighted in gray.

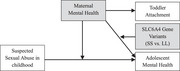

## 1 Introduction

Based on data from the Centers for Disease Control (CDC 2023), suicide rates exhibited a notable rise of approximately 36% during the period spanning from 2000 to 2021. In the year 2021, a total of 46,183 individuals tragically lost their lives to suicide, which is an average of one death occurring approximately every 11 min. Furthermore, it is noteworthy that in the year 2021, suicide emerged as one of the nine principal factors contributing to mortality among individuals ranging from 10 to 64 years of age. The CDC reports that suicide has emerged as the second most prominent cause of death among adolescents aged 10‐14 and young adults aged 20‐34 (CDC 2023). Suicide represents a significant public health concern that is pervasive in society, with a specific vulnerability observed among adolescents and young adults. While the etiology of suicide is multifaceted and lacks a singular causative factor, the National Institute of Mental Health (NIMH, 2023) lists several prominent risk factors, encompassing but not limited to depression, comorbid mental health conditions, and familial predisposition to mental disorders, and instances of physical or sexual abuse. Childhood sexual abuse showed the largest effect on increased risk for suicide attempts in adulthood compared to childhood physical and emotional abuse (Angelakis, Gillespie, and Panagioti 2019). Moreover, a systematic review of the research literature identified that sexual abuse is consistently linked to suicidal behavior (Serafini et al. 2015). In recent times, there has been a notable shift in attention towards incorporating maternal mental health into both suicide prevention research and practice, driven by the recognition of its consequential influence on the well‐being of the child (Wasserman et al. 2021). Maternal mental health has emerged as a significant public health concern in contemporary times, with a substantial increase in research funding and publication of studies from both developed and underdeveloped nations (Satyanarayana, Lukose, and Srinivasan 2011). Moreover, prenatal depressive and anxious symptomology is associated with child and adolescent emotional and behavioral issues (Leis et al. 2014) which could increase suicidal ideation risk.

Maternal mental health is complex and has observable stages of expression and influence on child development that could affect risks for suicidality. Antenatal depression has been linked with premature birth and disorganized attachment, while postnatal depression increases the risk of childhood and adolescent depression (Stein et al. 2014). Premature birth (Patton et al. 2004), depression (Grossberg and Rice 2023), and attachment issues have been linked to increased suicide risk in adolescence (Zortea, Gray, and O'Connor 2021). Most importantly, maternal mental illness appears to increase the severity of the mental illness presentation in the child (Rishel et al. 2006). Children and adolescents raised in a home where maternal mental health issues are present are directly impacted both immediately and in the long term in ways that set up a developmental trajectory that increases risk for suicidality in adolescence.

Attachment in early life is critical to development and mental health when considering suicide risk. The spectrum of effects on child and adolescent mental health ranges from suicide risk to protective of child development. The significance of healthy parental attachment was studied by Oldfield, Humphrey, and Hebron (2016), and the results demonstrated that a more secure parental attachment significantly correlated with lower levels of emotional difficulties, lower levels of conduct problems, and enhanced prosocial behavior. However, it is less clear how maternal depression and anxiety during the early life of a child may moderate the effects of attachment and childhood sexual abuse on risk factors of suicidal ideation such as self‐perceived depression and cognition associated with suicidal ideation.

It is important to also consider genetic factors that may play a role in shaping an adolescent's mental health in relation to their developmental experiences when examining the etiology of suicide risk. Numerous studies have provided evidence regarding the role of the serotonin transporter gene SLC6A4 in gene‐environment interactions across various developmental outcomes in both human and animal subjects. Specifically, SLC6A4 variants have been shown to moderate associations between adversity and physical development, behavior, and depression by increasing and decreasing risk for developmental outcomes according to the SLC6A4 variant. Wilson and Kinkead (2008) identified evidence to support the notion that the SLC6A4 gene plays a significant role in modulating the adverse effects of social subordination on the initiation of puberty in rhesus monkeys. More specifically, individuals who possess the short variant of the gene demonstrated a delay in their sexual development when exposed to conditions of subordination. Pubertal timing has been linked to increased adolescent mental health issues such as internalizing problems (Dehestani et al. 2023), which could increase suicide risk, although the associations have been shown to be sex‐specific. The SLC6A4 long/long allele has shown mitigating effects on the propensity for risky behavior among African American youths, aged approximately 11.2 years, who were enrolled in the Strong African American Families prevention program (Brody et al. 2009). The aforementioned phenomenon was noted during the comparison of these individuals with their counterparts who lacked the allele and were also participants in the program, as well as with peers who possessed and did not possess the allele but were not enrolled in the program. Furthermore, individuals with the genetic variation involving two short alleles of the SLC6A4 gene, combined with a history of childhood abuse, demonstrated an increased vulnerability to experiencing persistent depression in comparison to individuals without the two short alleles (Uher et al. 2011). These findings suggest that adolescent carriers of the SLC6A4 short/short variant with a history of childhood abuse may be at increased risk of suicide via genetic risk for persistent depression.

The potential impact of gene‐environment interactions on adolescents’ mental health and suicide‐related cognitions may be a mechanism of stress and helps to provide a framework for this study examining the suspected risk factors for suicidal ideation. The study conducted by Ollmann et al. (2021) discovered that the SLC6A4 gene plays a moderating role in the relationship between chronic stress and anxiety among individuals in the age range of 14 to 21 years. Numerous empirical investigations have provided evidence regarding the interplay between the SLC6A4 gene and stress‐inducing circumstances, resulting in an increased vulnerability to mental and behavioral health disorders (Ollmann et al. 2021). Mueller et al. (2012) proposed that the hypothesis suggested a correlation between specific variations of SLC6A4 and an individual's physiological response to psychological stress. The aforementioned concept bears importance in the examination of the ramifications of attachment and maternal mental health on adolescent mental health and cognitions associated with suicidal ideation risk. Specifically, attachment (Howard and Medway 2004, Cameron et al. 2017) and maternal mental health (Lengua et al. 2022) have been linked to adolescent stress sensitivity. Therefore, this study focused on the relationships between sexual abuse, toddler attachment (TA), maternal mental health, and adolescent mental health risks for suicidal ideation within the context of the SLC6A4 serotonin transport allele interactions.

## 2 Methods

### 2.1 Variables

Covariate and adolescent perceived mental health– Adolescent sex (Brent et al. 1999) and family poverty status (Dupéré, Leventhal, and Lacourse 2009) were selected for assessment of covariate effects given their prior associations with suicidal behavior. Moreover, poverty has been linked to stress and subsequent mental health and behavior in children (Wadsworth et al. 2008), making it a necessary covariate for analytical models. Poverty is negatively associated with adolescent DEP in multiple regression analysis (*B* = –1.923, *t* = –3.675, *p* = 0.001, bootstrap CI [–2.632, –1.228]) while controlling for suspected childhood sexual abuse and adolescent sex. Poverty was tested for associations with TA (*p* > 0.05), maternal anxiety (*p* > 0.05), and maternal depression, with only year one showing statistical significance (*B* = 0.063, *t* = 2.433, *p* = 0.015). Therefore, poverty was included as a covariate in all analyses for adolescent mental health. Adolescent sex did not associate with adolescent DEP or LNWL, so it was not included as a covariate in remaining analyses. The birth mother provided information regarding the sex of the child at the initial assessment (male or female), and further analysis was conducted to examine the potential influence of covariates. The presence of poverty was determined through the self‐reporting of household income by mothers, while the classification of poverty status was based on the federal poverty line for household income relative to the number of individuals residing in the family (coded as 0 for no poverty and 1 for poverty). The study assessed the perception of depression in adolescents using a self‐report measure of depressive feelings, “I feel depressed”, (DEP). Perceived (subjective) mental health has been linked to the use of mental health services more so than objectively measured mental health (Chiu et al. 2020) and may be a better indicator of suicide risk. Researchers recommend that one or two questions about self‐perceived depressed mood or anhedonia are sufficient to achieve 96% sensitivity and 57% specificity to detect major depression (Nakao et al. 2008). This may be due to a complex interplay of individual coping strategies and stress tolerance. Therefore, the authors elected to measure adolescent self‐perceived depression to demarcate adolescents who are subjectively experiencing depression from those who are not, regardless of objective observations that could be measured. Suicidal ideation risk is challenging to measure, as denial of suicidal thoughts is estimated at up to 46% of patients receiving mental health care who were directly asked about suicidal ideation (Blanchard and Farber 2020). Therefore, the authors elected to measure cognition associated with suicidal ideation risk that does not require admission of suicidal ideation. Adolescent suicidal ideation risk cognition was measured with self‐report of thoughts of life's worthlessness, “I feel life is not worth living”, (LNWL). The adolescent indication of “I feel life is not worth living” represents two cognitive markers, reasons for living and hopelessness, previously identified as risk factors for suicidal ideation and behavior (Wenzel and Beck 2008) and has been used in prior research (O'dwyer et al. 2014, Paykel et al. 1974, Lunsky 2004, Kirby et al. 1997). As a person's reasons for living reduce and their hopelessness increases, they become at greater risk for experiencing suicidal ideation and engaging in suicide behaviors (Wenzel and Beck 2008). Participants rated their agreement with these “I feel depressed” and “I feel life is not worth living” statements on a Likert scale ranging from 1 to 4, where higher scores (3 or 4) indicated agreement. Adolescent suicidal ideation risk cognition (I feel life is not worth living) did positively associate with adolescent self‐perceived depression (I feel depressed) while controlling for poverty status (*B* = 0.614, *t* = 28.879, *p* < 0.001), indicating support that these indicators of perceived mental health are related constructs.

Toddler attachment: Toddler attachment (TA) was evaluated with the standardized Attachment Q‐Sort measure (Waters and Deane 1985) used in research and practice to assess attachment in children aged 12‐48 months. The Q‐Sort method uses a set of cards to assess a child's behavior. A trained observer sorts the cards based on behavior descriptions, categorizing them into groups and ranking them. Rankings are compared to those of a securely attached child. A toddler's attachment is scored with a range of −1 to 1, with 1 indicating a perfectly securely attached child.

Maternal mental health: The presence of depression among mothers was assessed using the CIDI short form scale at three time points: year one (Mdep1: yes = 533), year three (Mdep3: yes = 607), and year five (Mdep5: yes = 486). The identification of depression was categorized as either positive (yes) or negative (no). Maternal depression at year 1 showed 38.46% of these mothers also tested positive for depression at year 3, and 28.70% tested positive for depression at year 5. Maternal depression at year 3 showed 33.77% of these mothers also tested positive for depression at year 5. The CIDI is a standardized scale found to have good reliability and validity (Andrews and Peters 1998). The CIDI short form scale was also utilized to evaluate the anxiety levels of mothers. This assessment determined whether or not the mother met the criteria for anxiety in both the first (Manx1: yes = 137) and third year (Manx3: yes = 193), with responses categorized as either “no” or “yes”. Maternal anxiety at year 1 showed 33.57% of these mothers also tested positive for anxiety at year 3.

Suspected child sexual abuse: The study assessed instances of suspected sexual abuse (SSA) by examining verified child protective services investigations pertaining to reported cases of SSA (“no”, “yes”) at three specific time points: when the children were 5 years old, 9 years old, and 15 years old.

Serotonin transporter gene (SLC6A4): This study examined the gene‐environment interactions by measuring two variables of the SLC6A4 serotonin transporter gene in the adolescents. For the evaluations, we opted for the short/short (SS) allele (negative or positive) and the long/long (LL) allele (negative or positive). We made the decision to eliminate alleles with variations (namely, S/L) that could potentially complicate the interpretation of our findings since extrapolating meaning from the S/L carrier associations is still in its infancy in research.

### 2.2 Hypotheses

This study aimed to test hypotheses in an effort to address the knowledge gap regarding the effects of maternal mental health, TA, and SSA in childhood on adolescent self‐perceived depression (DEP) and suicidal ideation risk cognition (LNWL). Additionally, gene‐environment interactions were examined to identify SLC6A4 variant moderating effects on direct environmental factor associations. The hypotheses are outlined as follows.

H1: Maternal mental health, anxiety (MANX), and depression (MDEP), will positively associate with adolescent self‐perceived depression (DEP) and suicidal ideation risk cognition (LNWL).
H2: TA will negatively associate with adolescent DEP and LNWL.
H3: SSA will positively associate with adolescent DEP and LNWL.
H4: Maternal mental health, MANX and MDEP, will moderate effects of TA and SSA on adolescent DEP and LNWL.
H5: SLC6A4 variants will moderate effects of MANX, MDEP, TA, and SSA on adolescent DEP and LNWL.

### 2.3 Sample

The data and sample utilized in this research were obtained from The Future of Families and Child Well‐being Study (McLanahan et al. 2003). The full cohort comprises a total of 4898 children and their corresponding caregivers, who were selected as a sample from major urban areas in the United States during the years 1998 to 2000. This study included 1829 participants from the full cohort with genetic data. The data collection process commenced by gathering initial information from the mothers shortly after the child's birth. Subsequently, follow‐up data was collected at various intervals as the children reached subsequent ages, specifically at 1, 3, 5, 9, 15, and 22 years. The data collection conducted between 1998 and 2000 involved conducting core interviews with both mothers and fathers upon the birth of the “focal child” in the study. Subsequent to the child's birth, interviews were conducted at the hospital. During the baseline and subsequent five waves, primary telephone interviews were conducted to gather data pertaining to parental relationships, parenting practices, health status and behaviors, family and social support systems, demographic characteristics, housing conditions, utilization of social programs, educational background, and employment status. The follow‐up wave conducted from 1999 to 2001 comprises core interviews with both mothers and fathers regarding the focal child when they reach their first birthday. The subsequent waves of data collection, specifically Year 3 (2001–2003) and Year 5 (2003–2006), encompassed interviews with both mothers and fathers, interviews with primary caregivers, and home visits conducted when the target child reached their third and fifth birthdays. The interviews with primary caregivers focused on topics related to health, daily routines, and parenting practices. During the period of Year 9 (2007–2010), the research protocol consisted of conducting core interviews with both the mother and father, as well as interviews with the primary caregiver. Additionally, house visits were conducted, and the interviewers made observations, similar to the procedures followed in the previous two waves. At approximately the age of nine, the primary child participant underwent an interview pertaining to topics such as family dynamics, school experiences, task fulfillment, self‐perception, and domestic routines. Saliva samples were obtained from the children of interest and their biological mothers during in‐home examinations to gather biomarker data, including telomere length and DNA methylation clocks. Additionally, genotyping and polygenic scores (PGS) were conducted. The collection of follow‐up data for Year 15 took place between the years 2014 and 2017. The data collection process involved conducting phone interviews with both the primary caregiver and the teenager, conducting home visits, and making observations during face‐to‐face interviews. The phone interviews encompassed various aspects related to the focal children, such as their educational background, experiences within the school environment, engagement in risky behaviors like sexual activity and substance use, interactions with peers, and involvement in pro‐social activities.

The child/adolescent cohort has a somewhat balanced distribution of gender, with males comprising 47.8% of the sample. Among the children, the largest ethnic group is African American (37.5%), followed by Latin American (16.5%), Anglo American/Caucasian (16.25%), Asian American (2.02%), Indigenous (1.08%), and 26.65% of children chose not to disclose their ancestral background. A significant proportion of the sample, specifically 68.7%, resided in households with an annual income that fell below the federal poverty threshold. Additionally, the average age of the mothers at the time of giving birth was 25.27 years (SD = 6.03). Variables included in the analyses have detailed descriptions in table 1.

**TABLE 1:** Variable descriptions.

Variable	Time point collected	Sample details
Household income: mean (SD)	Baseline	31,994.04 (31,567.17)
“I feel depressed”: mean (SD)	Year 15	1.522 (0.866)
“I feel life is not worth living”: mean (SD)	Year 15	1.249 (0.637)
Toddler attachment: mean (SD)	Year 3	0.757 (0.428)
Suspected sexual abuse	Year 5	Yes = 28
Maternal depression	Year 1 Year 3 Year 5	Yes = 533 Yes = 607 Yes = 486
Maternal anxiety	Year 1 Year 3	Yes = 137 Yes = 193
SLC6A4 variants	Year 9	SS = 451 LL = 1158

Abbreviations: SD is abbreviated for standard deviation.

### 2.4 Statistical Analysis

All analyses were conducted in SPSS version 29 (IBMCorp 2023).The variables employed in this study encompass multiple waves of data collecting derived from the longitudinal study known as The Future of Families and Child Wellbeing Survey. There was a lack of consistent engagement among participants across all variables and waves due to various reasons. Consequently, a missing value analysis was performed using Little's MCAR test, which revealed that the missing data was missing completely at random (*χ* ^2^ = 40.787, df = 28, *p* = 0.056). To account for missing data, the listwise deletion approach was employed for all analyses and resulted in a total sample of 1829 mother/adolescent dyads. The predictor and dependent variables were included in an initial correlation matrix to find any variables that were correlated and could be further analyzed using multiple regression analysis. Multiple regression was selected as the analytical approach given its consensus approval for moderation tests and ability to investigate complexity of multiple contributing factors influencing the dependent variables (Hayes 2017). Bootstrapping was used to reduce the risk of type I error caused by non‐normality of the data (Johnston and Faulkner 2021). Multiple comparisons were controlled with the Benjamini–Hockberg procedure for a false discovery rate of 15% [(individual *p*‐value rank/number of tests) * 0.15 (Benjamini and Hochberg 1995)]. A total of 16 multiple regression tests were conducted including the 6 moderation analyses and the BH adjusted *p*‐value threshold is reported with each statistically significant result. The study initially investigated the impact of maternal mental health, TA, and SSA, controlling for poverty status and adolescent sex, as key predictors on adolescent mental health. Subsequently, multiple regression analyses were conducted to explore the presence of moderation relationships between maternal mental health, TA, SSA, and serotonin transporter gene variants (SS/LL), and their link with adolescent mental health. The analytical conceptual figures are shown in Figure S1. This study was exempt from IRB review given the use of deidentified secondary data. All study procedures meet the ethical guidelines outlined in the Helsinki Declaration and Belmont Report.

## 3 Results

### 3.1 Maternal Mental Health, Suspected Childhood Sexual Abuse, and Poverty

SSA did not associate with adolescent DEP or adolescent LNWL when controlling for poverty status (*p* > 0.05). A multiple regression analysis was conducted to examine the relationship between maternal anxiety at years 1 (Manx1) and 3 (Manx3), and maternal depression at years 1 (Mdep1), 3 (Mdep3), and 5 (Mdep5) on TA. The results revealed that Mdep5 was significantly associated with reduced scores for TA (*B* = –0.072, *t* = –2.271, *p* = 0.023, BH *p*‐value threshold 0.028, bootstrap CI [–0.141, –0.002]). Manx1, Manx3, Mdep1, and Mdep3 did not associate with TA when controlling for Mdep5 (*p* > 0.05). Maternal depression and anxiety did not exert direct effects on adolescent agreement with LNWL or DEP (*p* > 0.05).

### 3.2 Maternal Mental Health and Toddler Attachment Interactions

TA was examined for direct effects on adolescent DEP and LNWL, and no associations reached statistical significance when controlling for poverty status (*p* > 0.05). Interaction variables were created with maternal mental health and TA and input into multiple regression models controlling for the independent terms (maternal mental health, TA) and poverty as a covariate. No moderating associations emerged statistically significant between Manx or Mdep and TA in relation to adolescent DEP or LNWL while controlling for poverty (*p* > 0.05). Poverty, however, was statistically significant in several of the models, and the direction of the relationship reversed from the direct effects of poverty when controlling for Manx, Mdep, and TA. Specifically, adolescents’ self‐perceived depression increased with positive indication of poverty status (see table 2) once the effects of maternal mental health and TA were statistically removed from the model.

**TABLE 2:** Maternal mental health and toddler attachment moderation with poverty covariate.

Predictor variable	Association	Outcome variable
(Manx1 X TA) + (Manx1) + (TA)	NS	Adolescent perceived depression
Poverty covariate	*B* = 0.09, t = 2.003, *p* = 0.045	
(Manx3 X TA) + (Manx3) + (TA)	NS	Adolescent perceived depression
Poverty covariate	*B* = 0.094, *t* = 2.107, *p* = 0.035	
(Mdep1 X TA) + (Mdep1) + (TA)	NS	Adolescent perceived depression
Poverty covariate	*B* = 0.088, *t* = 1.967, *p* = 0.049	
(Mdep3 X TA) + (Mdep3) + (TA)	NS	Adolescent perceived depression
Poverty covariate	*B* = 0.090, *t* = 2.021, *p* = 0.043	
(Mdep5 X TA) + (Mdep5) + (TA)	NS	Adolescent perceived depression
Poverty covariate	*B* = 0.110, *t* = 2.402, *p* = 0.016	

*Note*: Maternal mental health and toddler attachment variables are listed in one line to represent inclusion in the multiple regression model. NS indicates no statistical significance found. Poverty did not show statistical significance in any models examining associations with adolescent self‐report of cognition indicative of suicidality.

### 3.3 Suspected Sexual Abuse and Maternal Mental Health Interactions

**FIGURE 1:**
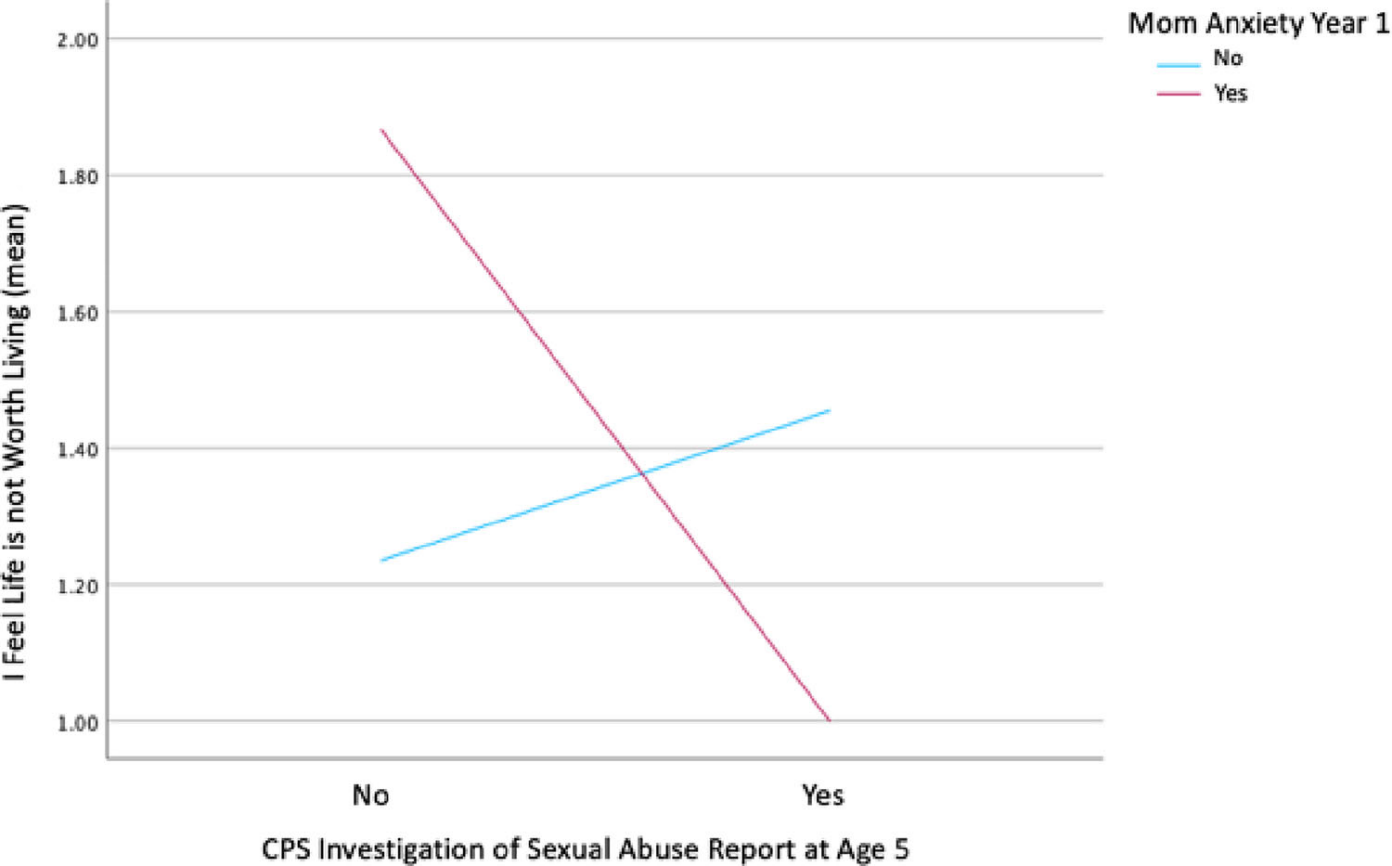
Maternal anxiety year 1 and suspected sexual abuse year 5 moderation.

Interaction variables were created with SSA and maternal mental health (Manx, Mdep) and input into multiple regression models controlling for the appropriate independent terms (SSA, maternal anxiety, maternal depression) and poverty as a covariate. SSA at years 5 and 15 were the only sexual abuse variables indicated in the correlation matrix with possible associations, so only those years were tested for interactions. SSA at year 5 and Manx1 showed a significant interaction effect of a moderation relationship in association with adolescent LNWL (*B* = –1.085, *t* = –2.429, *p* = 0.004, BH *p*‐value threshold 0.01, bootstrap CI [–1.730, –0.448]). Specifically, maternal anxiety at year one reduces self‐report of agreement with thoughts “Life is not worth living” in adolescents with SSA at age five years, as shown in Figure 1. SSA at year 15 and Mdep5 initially showed an interaction effect on adolescent LNWL, but the bootstrap confidence interval contained “0” and therefore did not support the association.

### 3.4 Serotonin Transporter Gene Interactions

Interaction variables were created with the serotonin transporter alleles (SS/LL) and maternal mental health, TA, and SSA and input into multiple regression models controlling for the independent terms and poverty as a covariate. The SLC6A4 LL variant moderated effects of maternal anxiety on adolescent suicide ideation risk cognition. Adolescents with the long/long allele who had mothers with anxiety at year one showed a higher mean score of agreement with “I feel life is not worth living,” shown in Figure 3 (*B* = 0.459, *t* = 2.954, *p* = 0.003, BH *p*‐value threshold 0.009, bootstrap CI [0.108, 0.955]). SLC6A4 variant interactions with maternal depression (*p* > 0.05), TA (*p* > 0.05), and SSA (*p* > 0.05) did not reach statistical significance with adolescent DEP or LNWL.

## 4 Discussion

This study was the first, to our knowledge, to investigate maternal mental health, TA, and SSA as key predictors of adolescent mental health within the context of SLC6A4 variant interactions. The analyses revealed four significant relationships: (1) poverty status is a strong predictor of adolescent mental health and must be included as a covariate, (2) TA is affected by maternal mental health but does not interact with maternal mental health to predict adolescent mental health, (3) maternal mental health affects suspected childhood sexual abuse associations with adolescent mental health, and (4) serotonin transporter alleles (SLC6A4) exert specific effects on adolescent mental health cognitions previously linked to suicide risk.

The findings indicated a significant negative relationship between poverty and adolescent DEP. While this study included poverty only as a covariate, the direct effects indicate poverty is a strong factor in the etiology of adolescent mental health and thereby risk for suicide. Individuals growing up in homes of poverty were more likely to report feeling depressed as adolescents. This finding supports the growing evidence that “poverty is both a cause of mental health problems and a consequence” (Knifton and Inglis 2020). Poverty overall affects an individual's mental health, and more specifically, has a clear relationship with mental health risks for suicide. In a study conducted in Scotland, suicides were three times more likely in deprived areas such as Glasgow (ISD 2019). With the significant increase in youth suicide deaths in the last couple of years, it would be imperative for future research to include poverty as a standard covariate for all adolescent mental health research and investigations examining risks for suicide. Moreover, there is a need for policy changes to provide additional and more diverse resources to aid those struggling socioeconomically as preventative measures.

**FIGURE 2:**
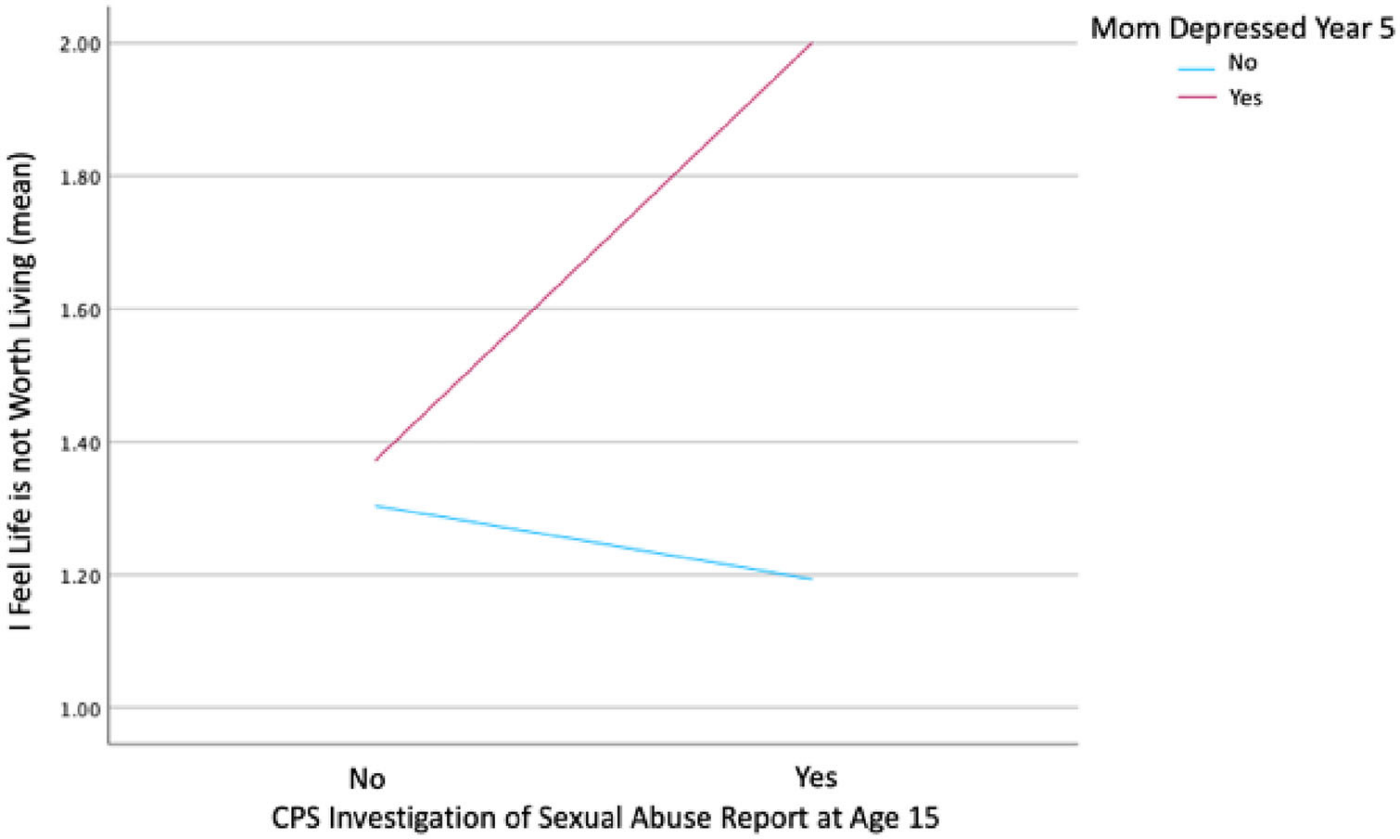
Maternal depression year 5 and suspected sexual abuse year 15. note: Interaction did not remain statistically significant with bootstrapping.

It is well documented that maternal depression is a risk factor for adolescent internalizing symptoms such as depression and anxiety (Monti and Rudolph 2017). In their study, Naicker, Wickham, and Colman (2012), found that adolescents who were initially exposed to maternal depression between the ages of 2‐5 years had a two‐fold increase in odds of developing an emotional disorder. Prior research has indicated maternal depression as a predictor of adolescent mental health and visual depictions of these variables suggest support of previous findings, albeit our approach did not meet statistical significance (see Figure 2 ). Contrary to previous research and our hypothesis, our findings revealed no direct effects of maternal mental health on adolescent self‐perceived depression (DEP) or self‐perceived suicidal ideation risk cognition (LNWL).

**FIGURE 3:**
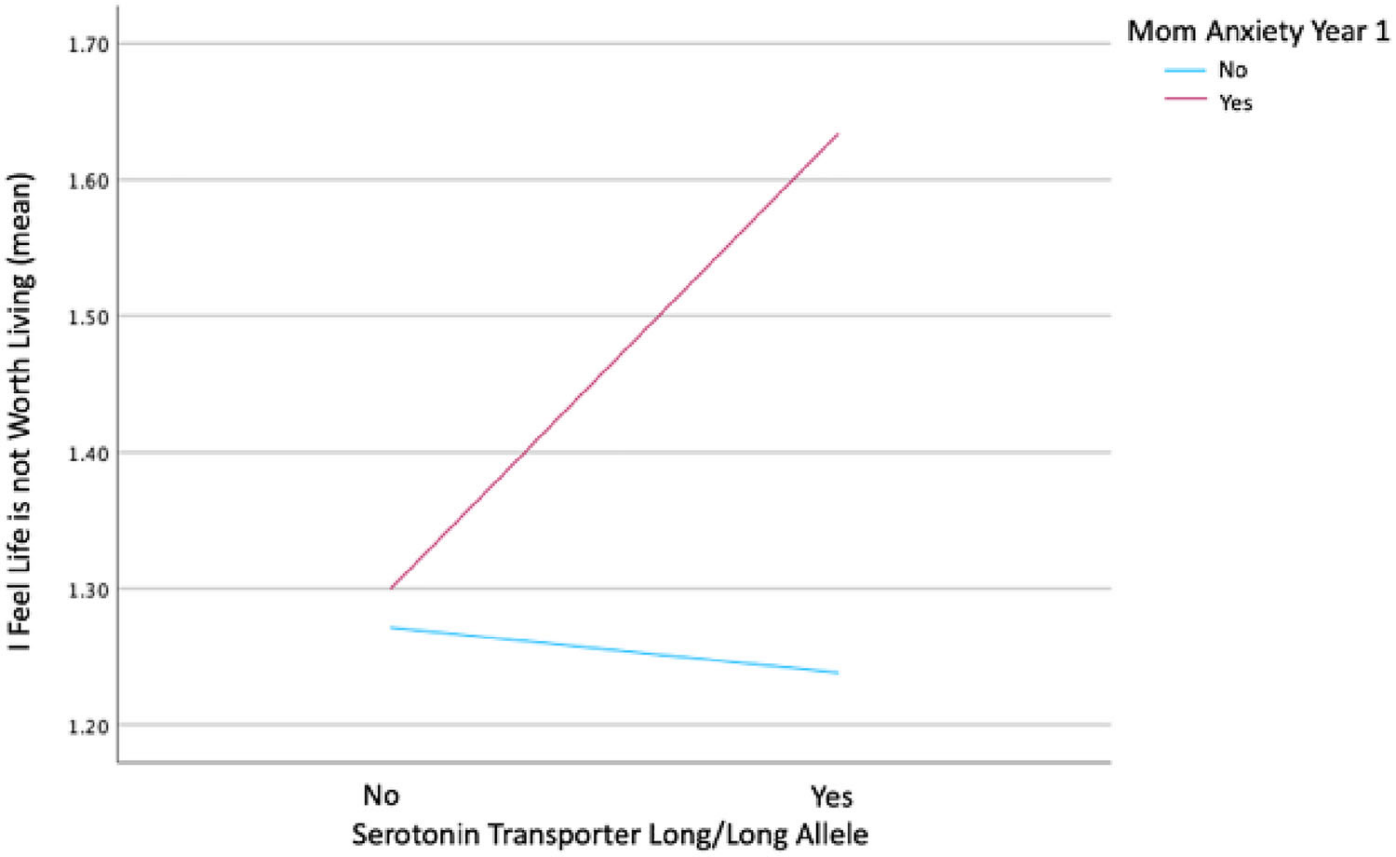
Serotonin transporter long/long allele and maternal anxiety year 1 moderation.

Furthermore, the interaction of maternal depression and adolescent SSA effects on adolescent internalizing suicidal ideation risk symptoms, specifically the self‐perceived agreement with the statement “I feel life is not worth living,” did not meet the significance threshold through bootstrapping in this study, contrary to our hypothesis. However, it is worth mentioning that the association would have been statistically significant if hypothesis testing had been the sole method of analysis. This does not imply that previous findings are less accurate, nor does it imply that our divergent findings obtained through bootstrapping are more accurate. The effectiveness of bootstrapping relies on the degree to which the study sample accurately represents the true population. There is a potential for the study sample to deviate from being representative of the actual population, either in an under representative or over representative manner. There is also a possibility that the measurement of adolescent mental health, when comparing objective measures to self‐reported measures, captures the abstract construct in distinct manners. Previous studies have identified variations in the associations with objective and perceived mental health (Chiu et al. 2020). Perceived mental health may capture a broader range of nuanced individual distress related to mental health, thereby facilitating the exploration of risk and resilience mechanisms in the context of mild to moderate mental health conditions. Moreover, we controlled for poverty status in all adolescent mental health analyses due to the large effect that emerged in testing for covariates. Poverty has been well linked to mental health (Tampubolon and Hanandita 2014, Lund 2014, Belle 1990, Goodman et al. 2013, Marbin et al. 2022), and it is possible that controlling for this covariate helps to distinguish between maternal mental health as the main effect versus possible causes of poor maternal mental health that also affect adolescent mental health. Nevertheless, it is crucial to consider these findings in light of the statistical methodologies and metrics employed, as is customary in any scholarly investigation.

This study confirmed one of our hypotheses and identified an interesting finding that generates more questions to be answered in future research. The results indicated that maternal anxiety at year one reduced agreement with thoughts of “Life is not worth living” in adolescents with SSA at age five, showing that maternal mental health does moderate effects of SSA on adolescent suicidal ideation risk cognition. It is possible that increased maternal anxiety affects child and adolescent perception of social support in a manner that protects from cognitive risks for suicide ideation. It is also possible that increased maternal anxiety affects the relationship dynamics between mother and child in a way that shifts emotional responsibility, often described as parentification of the child or adolescent (Van Loon et al. 2017), which could create a sense of responsibility in the adolescent to manage their own emotions in order to reduce distress in the mother. Maternal depression, not anxiety, did associate with reduced TA, but attachment did not have direct effects, nor did it interact with maternal mental health to exert effects on adolescent mental health or related cognition when controlling for poverty status. The absence of direct effects of TA and maternal mental health moderation of TA effects on adolescent mental health is in contradiction to our hypothesis. However, given the results, it is unlikely that attachment is involved in the unique finding with maternal anxiety, SSA, and adolescent mental health cognitions. Yet, it is clear that additional investigation is needed to parcel out the larger model with additional factors of perceived social support and parentification effects.

The emergence of the genetic variant effect in these findings underscores the intricate nature of human development and the process of adapting to the environment and confirms our hypothesis. The LL SLC6A4 allele interaction indicated increased agreement with cognition noted as a risk for suicidal ideation. In contrast, previous studies have frequently linked the SS allele with a heightened susceptibility to mental health risks (Rosenthal et al. 1998, Wilhelm et al. 2007, Heinz et al. 2007, Fratelli et al. 2020, Visser and Van Der Mast 2005). Nevertheless, certain research findings have suggested that individuals who possess the long/long allele may be at increased risk for some health outcomes. In a Korean cohort, individuals diagnosed with autism exhibited a higher prevalence of long/long allele carriers. (Cho et al. 2007). Furthermore, it was observed that young adults possessing the long/long allele did not exhibit sensitivity towards perceived social support as a driving factor for happiness within a collegiate population in Israel (Sheffer‐Matan et al. 2019). The protective nature of social support in mitigating suicide risk factors is widely recognized in academic literature (Thanoi et al. 2010, Sparks, Mitchell, and LeDuc 2023, Tedrus et al. 2023). It is possible that adversity, such as maternal mental health issues early in a child's life, generates consequences for LL carriers that are more salient due to a possible reduced sensitivity to external protective factors such as social support. It is also possible that genetic variation contributes to intergenerational transmission of mental health risk. It is important to note that our findings do not suggest a genetic predictor of mental health, but rather an interaction between one's genetic makeup and how they adapt to their experiences and environment. This is a critical distinction as we move further into the practice of precision medicine. Specifically, as we continue to identify genetic risk factors for outcomes associated with adversities such as poverty, maternal mental health, attachment, and sexual abuse, we can begin to identify protective factors that interact with genetic predispositions to foster resilience and health for children and adolescents. These prevention studies are on the horizon for social sciences, and the current work helps to contribute to those investigations. However, further investigation is still warranted to explore the interplay between maternal mental health, LL alleles in adolescents, and social support in the context of risk factors associated with suicidal thoughts and actions. This is important because the convergence of maternal anxiety, genetic predispositions, and the internalization of quality or worthiness of life during adolescence may render certain individuals more susceptible to mental health challenges and suicidal tendencies.

## 5 Limitations

It is important to consider the findings from this study within the context of some limitations. First, while the study has a large mother/adolescent dyad group (*n* = 1829), it is not the full cohort due to only a subset of the cohort having genetic variant data available. Therefore, without replication, these findings should not be generalized outside of the genetic subset of the cohort. Second, we elected to not include the SL carriers due to the complexity of trying to identify meaningful interpretations of possible associations for the mixed carriers. Previous research has grouped SLC6A4 variants into any “S” carrier, which included SS and S/L combined. However, it is unclear if the effects associated with SL carriers are due to the “S” or the “L” when trying to group in such a manner. While we selected SS and LL for the simplicity of interpretation based upon the underlying biological effects (SS carriers often show lower levels of serotonin), we do not know if SL carriers may also have gene‐environment interactions in relation to maternal mental health, TA, or SSA. It can be assumed that SL carriers are as common in the population as SS and LL carriers, suggesting that we examined a representative sample of only 2/3 of the population. Therefore, these findings cannot be generalized outside of the SS and LL carrier populations.

In summary, it can be concluded that both experiential factors and genetic variation have interconnected influences on the mental well‐being of adolescents. Previous research has established a connection between maternal mental health and poverty with regards to adolescent mental health. However, our findings indicate that poverty, even as a covariate, has a large, significant effect and is likely a precursor to maternal mental health in the theoretical framework of risk factors for adolescent mental health problems. Furthermore, the impact of SSA during childhood on the mental well‐being of adolescents is influenced by the presence of maternal mental health issues. Therefore, interventions aimed at addressing child trauma would be enhanced by incorporating a component that specifically addresses the mental well‐being of mothers. The authors declare no conflict of interest to report, and data from this study are available from the Future of Families and Child Well‐being Study.

## Author Contributions

**Stefanie Pilkay**: data curation, formal analysis, supervision, methodology, validation, resources, project administration. **Devorah Halevy**: conceptualization, investigation, writing–review and editing, resources, writing–original draft, methodology, visualization, formal analysis, project administration. **Danielle Femia**: writing–review and editing. **Sage Malina Holland**: writing–review and editing. **Stephanie Heiland**: writing–review and editing. **Andrew M Carroll**: writing–review and editing. **Sarah Nunes**: writing–review and editing. **Veerman, T**.: writing–review and editing. **Dudak, L**.: writing–review and editing.

### Peer Review

The peer review history for this article is available at https://publons.com/publon/10.1002/brb3.70247.

## Supporting information



Supporting Information

## Data Availability

The data that support the findings of this study are openly available in Princeton University at https://ffcws.princeton.edu/sites/g/files/toruqf4356/files/nationalreport.pdf.
