# Supplementary material for: Adolescent Depression and Cognition Risk for Suicide: An Investigation of Risk Factors and Gene Environment Interactions
Source: Brain Behav. 2025 Feb 5;15(2):e70247. doi: 10.1002/brb3.70247 (PMC11799590; doi:10.1002/brb3.70247)
Supplement: Supplementary file 1 — Supporting Information [file BRB3-15-e70247-s001.docx]

Supplemental Figure 1.

Analytical Conceptual Figures


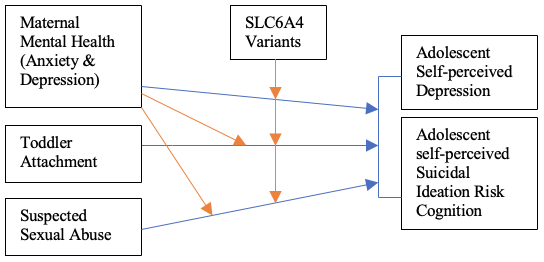


Note. Direct effect analyses are indicated with blue arrows and moderating analyses are marked with orange arrows.
